# Rethinking PINK1/Parkin-mediated mitophagy in Parkinson’s disease: functional continuity and activation–clearance uncoupling

**DOI:** 10.3389/fnagi.2026.1865383

**Published:** 2026-06-24

**Authors:** Lingzhe Wang, Bin Xu

**Affiliations:** 1 The Second School of Clinical Medicine, Zhejiang Chinese Medical University, Hangzhou, China; 2 Department of Neurology, The Second Affiliated Hospital of Zhejiang Chinese Medical University, Hangzhou, China

**Keywords:** functional uncoupling, lysosomal dysfunction, mitochondrial quality control, mitophagy, neuroinflammation, Parkin, Parkinson’s disease, PINK1

## Abstract

Mitochondrial dysfunction is a central feature of Parkinson’s disease (PD) and contributes to the selective vulnerability of nigral dopaminergic (DA) neurons. Among the pathways that maintain mitochondrial integrity, PINK1/Parkin-mediated mitophagy has been extensively characterized as a stress-responsive mechanism for the recognition and removal of damaged mitochondria. However, despite robust activation of this pathway in experimental systems, translation of these findings into effective disease-modifying strategies has remained limited. Here, we propose that a conceptual distinction may help account for this gap. Current research has largely focused on pathway activation as a surrogate for functional recovery, yet mitochondrial quality control depends on the maintenance of functional continuity across multiple sequential steps, from damage recognition and ubiquitin signaling to autophagosome formation and lysosomal degradation. Disruption at any of these stages may compromise overall pathway output. Accumulating evidence suggests that, under PD-relevant conditions, upstream signaling and downstream mitochondrial clearance can become partially uncoupled, such that activation of the PINK1/Parkin pathway does not necessarily ensure effective completion of mitophagy. Within this framework, mitochondrial dysfunction interacts with *α*-synuclein (α-syn) accumulation, lysosomal impairment, and neuroinflammatory signaling to form a self-reinforcing pathological network. This perspective provides a mechanistic basis for understanding why strategies that enhance upstream signaling alone have shown limited translational success. Finally, we discuss key challenges for therapeutic development, including the need for readouts that distinguish pathway engagement from pathway completion, the limitations of current model systems, and the importance of aligning patient stratification and intervention timing with pathway biology. We suggest that restoring functional continuity across the mitophagic process, rather than focusing exclusively on increasing pathway activation, may offer a more productive conceptual basis for targeting mitochondrial dysfunction in PD.

## Graphical abstract

**Figure:**
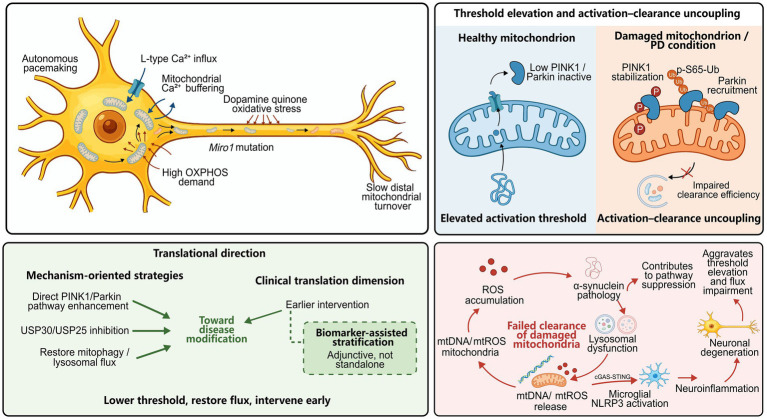

## 1 Introduction

Parkinson’s disease (PD) is among the fastest growing neurodegenerative disorders worldwide (Uoselis et al., 2023; Ben-Shlomo et al., 2024). Current treatment remains largely centered on dopaminergic (DA) replacement, which can provide symptomatic benefit but does not halt the progressive degeneration of DA neurons in the substantia nigra pars compacta (SNc; Antico et al., 2025). A central challenge in PD research, therefore, is to identify disease-modifying targets that can slow or prevent this neuronal loss (Hong et al., 2024; Foltynie et al., 2026).

DA neurons are particularly susceptible to mitochondrial dysfunction because several intrinsic biological properties converge to place exceptional stress on mitochondrial homeostasis (Kumar et al., 2017). Their autonomous pacemaking activity sustains continuous Ca^2+^ cycling, their energy demands rely heavily on persistent oxidative phosphorylation, and their highly elaborate axonal arbor requires mitochondrial trafficking over long distances (Zampese et al., 2022). Under these conditions, mitochondrial quality control is not simply supportive but essential for neuronal survival (Ahmed et al., 2026). Mitophagy, as the principal pathway for the selective removal of damaged mitochondria, is therefore considered an important mechanism for limiting oxidative injury, preserving mitochondrial homeostasis, and maintaining neuronal function (Xu et al., 2025).

Among the mitochondrial quality-control pathways implicated in PD, PINK1/Parkin-mediated mitophagy has received sustained attention in both genetic and mechanistic studies (Narendra and Youle, 2024). Yet the translational record of this pathway remains disappointing: interventions that appear neuroprotective in cellular and animal models have proven far more difficult to translate into clinically meaningful benefit. One reason is that many studies emphasize whether upstream pathway signals can be induced, while paying much less attention to whether such activation actually restores mitochondrial quality control in a functional sense (Ham et al., 2025). In PD, failure of the PINK1/Parkin axis may not be adequately captured as a single uniform defect. Instead, accumulating evidence suggests that dysfunction may arise at multiple levels, including impaired threshold crossing at the stage of pathway initiation and a possible dissociation between upstream signal amplification and downstream autophagic execution.

This review therefore examines PINK1/Parkin pathway imbalance in PD from the perspective of functional failure across the mitophagic cascade. We first outline the molecular logic that governs pathway activation and execution, then discuss why this system becomes particularly fragile in DA neurons, and finally evaluate how this mechanistic imbalance shapes current therapeutic and translational strategies. By focusing on the gap between pathway activation and effective mitochondrial clearance, we aim to clarify a central obstacle in the development of mitochondria-targeted interventions for PD.

## 2 Molecular logic of the PINK1/Parkin pathway: from activation to clearance

### 2.1 The resting state: setting the activation threshold

Under basal conditions, PINK1 is constitutively imported into mitochondria through the TOM and TIM23 translocase complexes (Akabane et al., 2023), where it undergoes sequential proteolytic processing by mitochondrial processing peptidase (MPP) and the inner membrane protease PARL (Jin et al., 2010). The resulting cleaved fragment is then released into the cytosol and rapidly degraded (Narendra et al., 2010), preventing its stable accumulation on the outer mitochondrial membrane (Deas et al., 2011). At the same time, cytosolic Parkin is maintained in an autoinhibited state in which the ubiquitin-like domain constrains the activity of the RING-between-RING (RBR) catalytic core (Sauvé et al., 2015; Kumar et al., 2017; Clausen et al., 2024). Together, these processes keep the pathway in a low-responsiveness state under physiological conditions.

**Figure 1:**
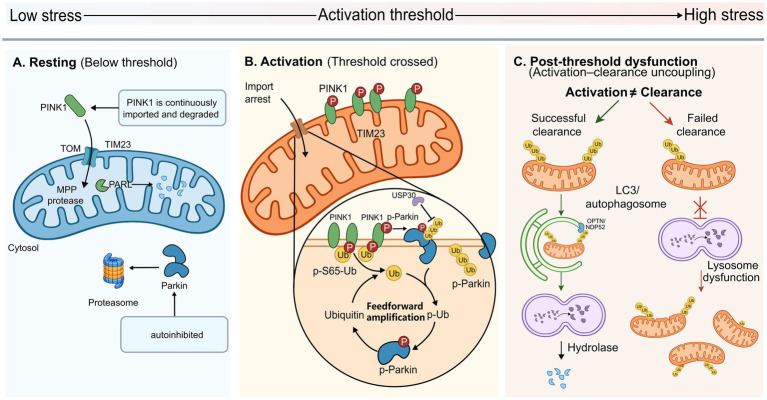
Mechanistic framework of PINK1/Parkin-mediated mitophagy, including activation, signal amplification, and execution failure. **(A)** Resting state: Under basal conditions, PINK1 is continuously imported and degraded, preventing its accumulation on the outer mitochondrial membrane. Parkin remains autoinhibited in the cytosol. **(B)** Activation: Mitochondrial stress blocks PINK1 import, leading to its stabilization and autophosphorylation. PINK1 phosphorylates ubiquitin (p-S65-Ub) and activates Parkin, triggering ubiquitination of mitochondrial substrates and feed-forward signal amplification. **(C)** Post-threshold dysfunction: Activation of upstream signaling does not necessarily result in mitochondrial clearance. While damaged mitochondria can be targeted to autophagosomes, impaired lysosomal degradation leads to accumulation of ubiquitinated mitochondria, reflecting activation–clearance uncoupling.

This basal state should not be viewed as complete pathway quiescence. Rather, it defines an activation threshold that must be exceeded before mitophagy can be productively engaged. Minor perturbations in mitochondrial membrane potential are insufficient to trigger this transition. By contrast, when mitochondrial damage disrupts import through TIM23, PINK1 is no longer delivered for proteolytic turnover and instead accumulates on the outer mitochondrial membrane (Michaelis et al., 2022; Eldeeb et al., 2024; Thayer et al., 2025). In this way, the resting state functions as a regulatory gate that determines when mitochondrial stress is severe enough to initiate downstream signaling (Figure 1A).

### 2.2 Damage recognition and signal amplification

Once mitochondrial damage exceeds this threshold, PINK1 is stabilized on the outer mitochondrial membrane and undergoes autophosphorylation (Akabane et al., 2023; Fiesel et al., 2023), thereby converting a local damage signal into a signaling-competent state (Okatsu et al., 2012; Kakade et al., 2022). PINK1 then phosphorylates ubiquitin at Ser65, generating p-S65-Ub, which acts as the central signal for Parkin recruitment and activation (Kane et al., 2014). Phosphorylation of Parkin’s Ubl domain further relieves its autoinhibited conformation and promotes activation of its E3 ubiquitin ligase activity (Ordureau et al., 2014; Lenka et al., 2024; Sauvé et al., 2024).

Activated Parkin ubiquitinates multiple outer mitochondrial membrane substrates, including mitofusins, thereby generating additional ubiquitin moieties that can themselves be phosphorylated by PINK1 (Kane et al., 2014; Ordureau et al., 2014). This creates a feed-forward amplification loop in which local accumulation of p-S65-Ub progressively reinforces Parkin recruitment and ubiquitination on the mitochondrial surface (Fakih et al., 2023; Sauvé et al., 2024). Although stress-responsive pathways such as AMPK/ULK1 may influence the sensitivity of this process, p-S65-Ub remains the central node that links damage recognition to sustained signal amplification (Sarraf et al., 2013; Lazarou et al., 2015; Vranas et al., 2022; Lu et al., 2026; Figure 1B).

### 2.3 From signal amplification to functional clearance

Upstream activation of the PINK1/Parkin pathway does not by itself ensure the removal of damaged mitochondria (Lazarou et al., 2015). Once ubiquitin labeling has been established on the mitochondrial surface, successful mitophagy requires coupling of this signal to downstream autophagic machinery (Heo et al., 2015). Autophagy receptors such as OPTN, NDP52, and TAX1BP1 recognize ubiquitinated mitochondrial substrates and link them to LC3/GABARAP-positive membranes, thereby initiating sequestration by the forming autophagosome (Lazarou et al., 2015; Le Guerroué et al., 2023; Meyer et al., 2023). TANK-binding kinase 1 (TBK1) further strengthens this process by phosphorylating receptors such as OPTN and enhancing their affinity for both ubiquitin chains and LC3 family proteins (Richter et al., 2016; Yamano et al., 2024).

Even with robust upstream signaling, however, completion of the pathway remains contingent on efficient progression through autophagosome maturation and lysosomal degradation (Tofaris, 2012). This is one reason why experimental readouts such as PINK1 stabilization, Parkin translocation, or p-S65-Ub accumulation cannot be assumed to indicate effective mitochondrial clearance. They capture activation of the damage-recognition program, but not necessarily successful completion of the degradative process.

Negative regulation further shapes this transition. USP30, which resides on the outer mitochondrial membrane, counteracts Parkin-mediated ubiquitination and restricts the substrate pool available for further PINK1-dependent phosphorylation (Fang et al., 2023). In parallel, defects in autophagosome maturation or lysosomal competence can interrupt the pathway after signal amplification has already occurred (Qu et al., 2022). The PINK1/Parkin pathway therefore operates as a multi-step system in which damage sensing, signal amplification, and terminal degradation are only partially coupled (Waters et al., 2023). This functional organization provides the mechanistic basis for the divergence between upstream pathway activation and effective mitochondrial clearance (Figure 1C).

## 3 Clinical and model-based evidence for PINK1/Parkin pathway dysfunction in PD

Evidence for PINK1/Parkin pathway dysfunction in PD derives from clinical biospecimens and experimental model systems, which capture different aspects of the same regulatory process (Hou et al., 2023). Clinical studies primarily report pathway-associated signals under disease conditions, whereas model systems define the functional consequences of pathway disruption (Narendra and Youle, 2024). Interpreting these datasets together therefore requires distinguishing between observable molecular outputs and effective mitochondrial quality control (Fiesel et al., 2025).

The clinical evidence summarized in Table 1 and integrated in Table 2 is consistent with the disease relevance of PINK1/Parkin-associated mitochondrial quality control in Parkinson’s disease, though these findings remain inherently indirect. Current studies rely largely on molecular signals detected in peripheral biofluids or postmortem tissue and therefore cannot directly determine whether altered pathway-associated markers correspond to impaired mitochondrial clearance within vulnerable nigral DA neurons. As outlined in Table 2, similar biomarker patterns may emerge from multiple biological processes, including pathway activation, compensatory responses, impaired degradation, or disease-related neuronal loss. Clinical findings therefore support disease relevance and provide a basis for testable predictions consistent with the activation–clearance framework (Hou et al., 2018; Watzlawik et al., 2021; Hong et al., 2024; Fiesel et al., 2025; He, H. et al., 2025).

**Table 1:** Clinical evidence for dysregulation of the PINK1/Parkin pathway in patients with PD.

Biospecimen source	Biomarker (direction of change)	Study cohort (*n*)	Reference
Brain tissue	p-S65-Ub ↑ (in Lewy body-associated neurons)	HC (*n* = 15); LBD (*n* = 9); LBD-SNCA (*n* = 6); MSA-P (*n* = 15)	Hou et al. (2023)
CSF/Plasma	p-S65-Ub ↓ (CSF); ≈ (plasma)	Plasma (*n* > 1,500); CSF (*n* = 149)	Fiesel et al. (2025)
Plasma	Parkin ↑	PD (*n* = 197); HC (*n* = 107)	He et al. (2025a)
	PINK1 ↑	PD (*n* = 197); PDS (*n* = 50); HC (*n* = 107)	Hong et al. (2024)
	PINK1 ↑; Parkin ↑; PGAM5 ↑	PD (*n* = 179); PDS (*n* = 90); HC (*n* = 116)	Qian et al. (2024)

↑, Elevated relative to healthy controls; ↓, reduced relative to healthy controls; ≈, no significant difference. HC, healthy controls; LBD, Lewy body disease; LBD-SNCA, Lewy body disease with SNCA pathology; MSA-P, multiple system atrophy–Parkinsonism; CSF, cerebrospinal fluid; PD, Parkinson’s disease; PDS, Parkinsonism syndrome.

**Table 2:** Evidence base underlying the activation–clearance uncoupling framework.

Evidence domain	Representative observation	Relevance to uncoupling framework	Strength*	Key limitation	Reference
Acute cell systems	PINK1 stabilization, Parkin recruitment, p-S65-Ub accumulation without proportional mitochondrial removal	Direct support	High	Acute depolarization paradigms	Narendra et al. (2008) and Heo et al. (2015)
Genetic/cellular PD models	Impaired downstream turnover despite preserved pathway activation	Direct support	Moderate–High	Artificial genetic context	Fakih et al. (2023) and Hou et al. (2023)
Human brain tissue	Altered p-S65-Ub-positive mitochondrial structures	Indirect support	Moderate	End-stage tissue; static snapshot	Hou et al. (2018) and Watzlawik et al. (2021)
CSF/plasma biomarkers	Divergent PINK1/Parkin/p-S65-Ub findings across biological compartments	Indirect support	Moderate	Peripheral signal ambiguity	Hong et al. (2024) and Fiesel et al. (2025)
Dynamic flux assays	mito-QC, mt-Keima, turnover kinetics distinguish initiation from completion	Validation strategy	High	Limited application in human PD samples	McWilliams et al. (2018b) and Morinaga et al. (2024)

* Strength reflects the extent to which a given evidence domain can distinguish pathway activation from effective mitochondrial clearance, rather than overall study quality.

As summarized in Table 1, alterations in PINK1/Parkin-related markers are consistently reported in PD, but their patterns vary across biological compartments. Brain tissue studies tend to show increased mitochondrial ubiquitin signaling, consistent with local activation of damage-responsive processes. In contrast, plasma measurements frequently report elevated levels of PINK1 and Parkin, while CSF findings are less consistent. These differences are unlikely to reflect experimental variability alone. Rather, they indicate that distinct biospecimens capture different projections of pathway activity, including local mitochondrial labeling, systemic stress responses, and potentially altered downstream processing (Baninameh et al., 2025).

This compartment-dependent divergence has an important implication. It suggests that clinical readouts of the PINK1/Parkin pathway should be interpreted as context-dependent outputs, rather than as direct measures of mitophagic efficiency (Fiesel et al., 2025). Increased detectability of pathway components—facilitated by advances in assay sensitivity—does not resolve this limitation (Baninameh et al., 2025). Because PINK1 is normally maintained at low steady-state levels through continuous import and degradation, its measurable accumulation may reflect multiple underlying states, including pathway activation, impaired downstream flux, or release from damaged cells (Jin et al., 2010). As a result, biomarker elevation alone cannot distinguish between effective mitochondrial clearance and incomplete pathway execution (Uoselis et al., 2023).

In contrast, the studies summarized in Table 3 provide evidence for the functional consequences of disrupting the PINK1/Parkin pathway. Across experimental models, loss of pathway function consistently leads to impaired mitochondrial quality control, defective ubiquitin signaling, and accumulation of dysfunctional mitochondria, ultimately promoting DA neurodegeneration. These findings demonstrate that pathway integrity is required for effective mitochondrial clearance and establish a direct link between pathway failure and disease-relevant phenotypes.

**Table 3:** Representative evidence of PINK1/Parkin pathway dysfunction in experimental models of PD.

Experimental model	Model features	Key findings	Reference
Non-human primate model	Rhesus macaque *PRKN*-deficient model	TH^+^ DA neurons in SNc ↓; pS129-α-syn accumulation ↑	Han et al. (2024a)
Rodent model	*Pink1*^−/−^ rats, 3 months of age; prodromal whole-blood transcriptome model	Peripheral inflammatory signaling ↑; interferon pathway activity ↑	Lechner et al. (2024)
	8-month-old P1KO rats; gait–cerebellar circuit model	TH^+^ neurons in SNc ↓; cerebellar neuroelectrical activity ↓; gait abnormalities ↑	DeAngelo et al. (2024)
Insect model	Drosophila *Pink1/PRKN* mutant flies	Mitophagic flux ↓; neurodegenerative phenotype ↑; phenotype partially rescued by Cisd inhibition	Martinez et al. (2024)
Primary cell model	Parkin-mutant skin fibroblasts derived from PD patients	Mitochondrial network complexity ↓; mitochondrial biogenesis-related proteins ↓; PINK1/Parkin pathway-associated proteins ↓	Kamienieva et al. (2023)
Cell model	hiPSC-derived midbrain DA neurons from patients with biallelic *PRKN* exon 2 deletion	ParkinΔ1–79 partially retains mitochondrial translocation capacity; E3 ubiquitin ligase activity partially retained; mitophagy initiation capacity partially retained	Hach et al. (2026)

↑, Elevated, increased, or enhanced; ↓, reduced, decreased, or impaired. TH, tyrosine hydroxylase; pS129-α-syn, α-synuclein phosphorylated at serine 129; P1KO, PINK1 knockout; PRKN, Parkin gene; hiPSC, human induced pluripotent stem cell.

When considered together, the evidence from Tables 1, 3 reveals a consistent pattern. Clinical studies detect pathway-associated signals in PD, whereas model systems show that disruption of the pathway results in defective mitochondrial turnover (Narendra and Youle, 2024). These observations are not necessarily contradictory. Instead, they may reflect different readouts of a system in which upstream activation and downstream clearance can become only partially coupled under disease-relevant conditions.

## 4 Physiological constraints of DA neurons and system-level imbalance of the PINK1/Parkin pathway in PD

### 4.1 Convergent physiological pressures define a high-risk regime for mitochondrial quality control

Nigral DA neurons operate under a set of physiological constraints that place mitochondrial quality control in a regime of unusually high demand (Liu et al., 2026). Rather than acting independently, these constraints interact to shape a system in which mitochondrial damage is continuously generated, spatially distributed, and energetically costly to resolve.

A primary driver of this state is autonomous pacemaking activity, which imposes sustained Ca^2+^ influx and requires continuous mitochondrial buffering (Zampese et al., 2022). This calcium-dependent workload is coupled to high rates of oxidative phosphorylation, such that energy production and calcium handling are not separable processes but mutually reinforcing demands on mitochondrial function (Liu et al., 2026). Under these conditions, increases in respiratory flux are intrinsically linked to elevated ROS production, creating a baseline level of oxidative stress that must be actively managed.

This metabolic burden is further amplified by neuronal architecture. The exceptionally elaborate axonal arbor of DA neurons requires mitochondria to be transported over long distances and maintained at distal synaptic sites, where local turnover is comparatively slow (Nadtochy et al., 2025). As a result, mitochondrial damage is not only generated but also spatially constrained, making timely removal more difficult. Disruption of this transport system, as observed in PD-associated Miro1 mutations, illustrates how impaired mitochondrial trafficking can convert physiological demand into selective neuronal vulnerability (Zagare et al., 2025).

In parallel, dopamine metabolism introduces an additional, cell-type-specific source of mitochondrial stress (Jana et al., 2011). Cytosolic dopamine readily undergoes auto-oxidation, generating reactive intermediates such as dopamine quinone that directly damage mitochondrial components and further amplify oxidative burden (Lee et al., 2025). Because post-mitotic neurons cannot dilute damaged organelles through cell division, these convergent pressures accumulate over time (Batlevi and La Spada, 2011).

**Figure 2:**
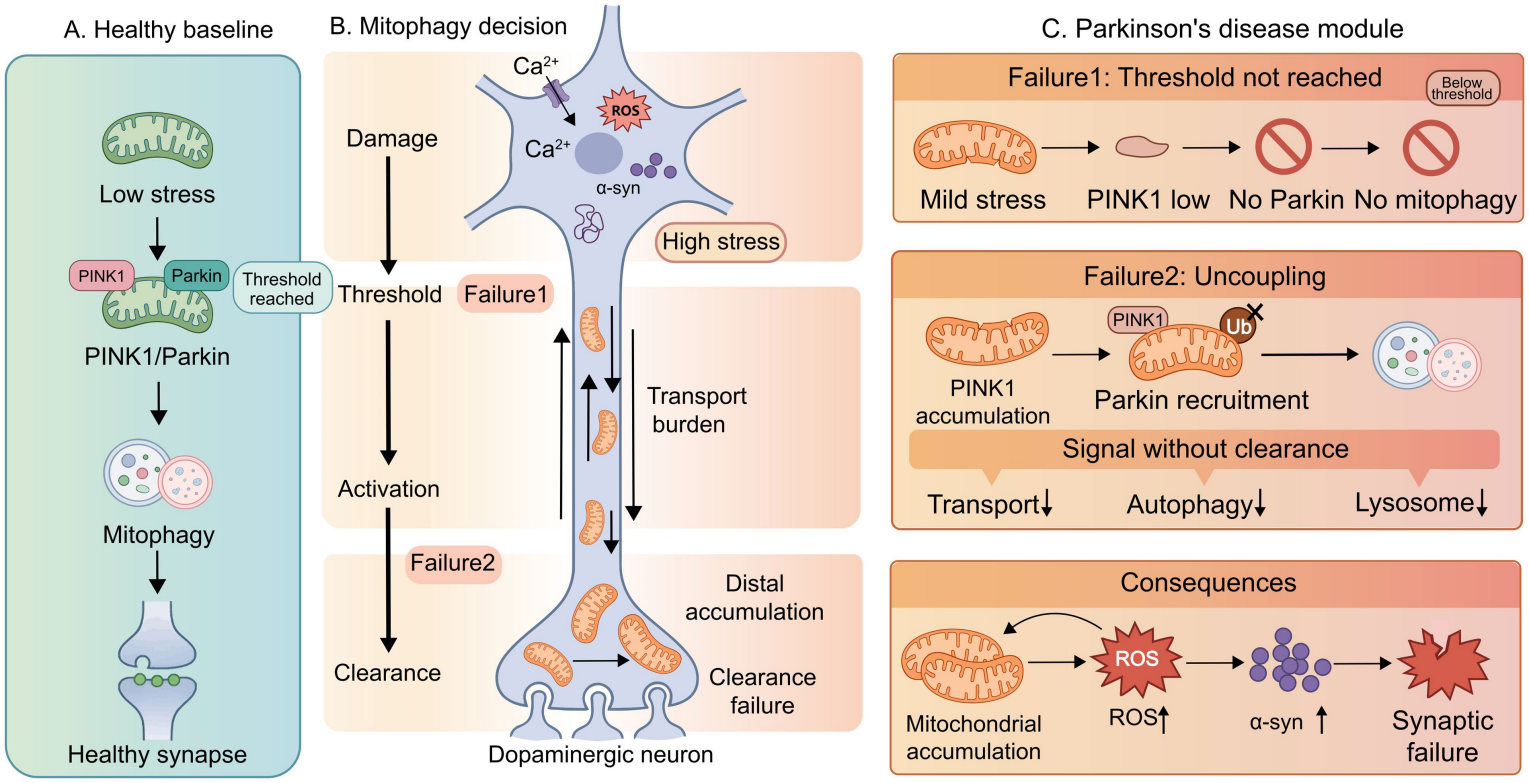
Physiological vulnerability of DA neurons and dual failure modes of PINK1/Parkin-mediated mitophagy in PD. **(A)** Healthy baseline: Nigral DA neurons experience sustained mitochondrial stress due to autonomous pacemaking-associated Ca^2+^ influx, high oxidative phosphorylation demand, dopamine-related oxidative stress, and slow distal mitochondrial turnover. Impaired mitochondrial transport further increases this vulnerability. **(B)** Mitophagy decision: Under pathological conditions, suppression of PINK1 activity raises the activation threshold of the PINK1/Parkin pathway, limiting mitophagy initiation (Failure 1). Even when upstream activation is achieved, downstream clearance may remain incomplete because of defects in transport, autophagy, or lysosomal degradation, resulting in activation–clearance uncoupling (Failure 2). **(C)** Parkinson’s disease module: Threshold elevation and activation–clearance uncoupling contribute to mitochondrial accumulation, ROS production, *α*-syn pathology, and synaptic dysfunction, forming a self-reinforcing cycle of neuronal degeneration.

From a systems perspective, these features place DA neurons in a regime in which mitochondrial quality control is not a background maintenance process but a continuously engaged requirement. In such a regime, the PINK1/Parkin pathway functions as a threshold-dependent decision system: it must discriminate between tolerable fluctuations and damage states that require organelle elimination (Waters et al., 2023). When damage generation is persistent and spatially distributed, this decision process is repeatedly challenged, increasing the likelihood that threshold crossing becomes inefficient or delayed. Under these conditions, even modest impairments in pathway activation or execution can disproportionately affect mitochondrial turnover, thereby sensitizing DA neurons to cumulative dysfunction (McWilliams et al., 2018b; Figure 2A).

### 4.2 Distinct entry points and differential evidence for PINK1/Parkin pathway dysfunction in familial and sporadic PD

In familial PD, mutations in *PINK1* or *PRKN* disrupt the core architecture of this decision system (Ando et al., 2017). These mutations may impair PINK1 stabilization, reduce kinase activity, or compromise Parkin’s E3 ligase function, thereby weakening the generation and amplification of phosphorylated ubiquitin signals (Connelly et al., 2024; Wagner et al., 2025; Figure 2C). In systems terms, such defects lower the efficiency of signal propagation and increase the effective threshold required to trigger a productive mitophagic response (McWilliams et al., 2018a; Fakih et al., 2023; Watzlawik et al., 2024a). As a result, mitochondria that would normally cross the activation threshold may fail to do so, or do so only weakly, leading to insufficient labeling and delayed clearance.

Sporadic PD, by contrast, more often reflects a pathological shift in the cellular environment that alters how this decision system operates (Oh et al., 2017; Rosencrans et al., 2025). Chronic oxidative stress, *α*-synuclein (α-syn) accumulation, and inflammatory signaling can interfere with pathway responsiveness through post-translational modifications and signaling interference (Shen and Dettmer, 2024). Rather than removing pathway components, these processes reduce their functional efficiency, effectively raising the activation threshold and dampening signal amplification (Figure 2C).

Despite operating through distinct primary mechanisms, both familial and sporadic forms of PD may converge on qualitatively similar disruptions of PINK1/Parkin-dependent mitochondrial surveillance, although the strength of evidence for this convergence differs substantially between the two disease contexts. In each case, mitochondrial damage may be generated at a rate that exceeds the capacity of the PINK1/Parkin system to respond effectively (Wang et al., 2023). This mismatch can manifest in two non-exclusive ways: failure to reach the activation threshold under conditions of chronic stress, and incomplete coupling between upstream signal amplification and downstream mitochondrial clearance. The proposed result is a progressive accumulation of dysfunctional mitochondria, which may feed back into oxidative stress, metabolic imbalance, and neuronal vulnerability.

The strength of evidence differs substantially between genetic and sporadic forms of PD. As summarized in Table 2, direct mechanistic support for impaired PINK1/Parkin-associated mitochondrial quality control derives primarily from genetic PD, where pathogenic variants affecting PINK1, Parkin, and related pathways link defective mitochondrial surveillance to neuronal dysfunction and degeneration (Ando et al., 2017; Clausen et al., 2024; Han et al., 2024b). In contrast, evidence in sporadic PD is drawn largely from biomarker studies, postmortem observations, and *α*-synuclein-associated models summarized in Tables 1, 2. Accordingly, Activation–Clearance Uncoupling is treated here as a conceptual framework for integrating available evidence and generating testable hypotheses across both disease contexts, rather than as a validated pathogenic mechanism in either form of PD.

## 5 Pathological network interactions of PINK1/Parkin pathway imbalance in PD

**Figure 3:**
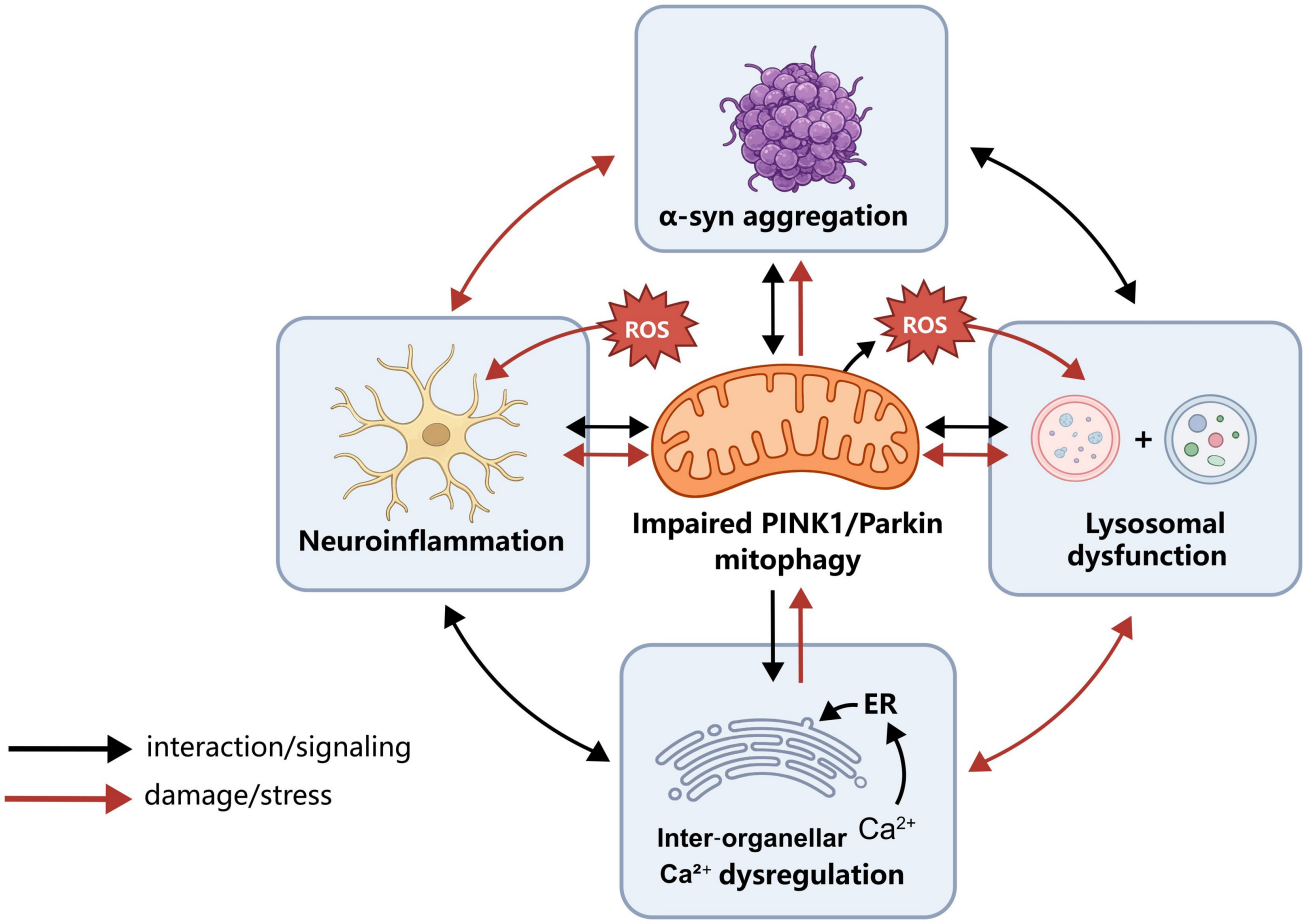
Self-amplifying pathological network driven by mitochondrial dysfunction in Parkinson’s disease. Mitochondrial dysfunction increases reactive oxygen species (ROS) production, which promotes α-syn aggregation and contributes to lysosomal impairment. Defective lysosomal function further exacerbates mitochondrial damage. In parallel, mitochondrial stress activates neuroinflammatory responses, which feeds back to worsen mitochondrial dysfunction. Inter-organellar Ca^2+^ dysregulation further amplifies this cycle. These interconnected processes form a self-reinforcing network driving progressive neuronal degeneration.

Impairment of the PINK1/Parkin pathway is not an isolated event; rather, it may contribute to a self-reinforcing pathological network in which impaired mitochondrial clearance becomes progressively coupled to cellular stress amplification (Sironi et al., 2020; Pereira et al., 2023; Narendra and Youle, 2024; Figures 2C, 3).

### 5.1 Α-Syn aggregation and the PINK1/Parkin pathway: a coupled Proteotoxic Axis

Impaired mitophagy leads to the accumulation of dysfunctional mitochondria, which act as a persistent source of reactive oxygen species (ROS). Elevated ROS promotes oxidative modification of α-syn, increasing its propensity to misfold and aggregate (Madsen et al., 2021). These observations suggest a mechanistic connection between mitochondrial dysfunction and α-syn pathology, though the relative contribution of this relationship in human disease settings remains to be fully characterized.

The reverse interaction is mechanistically distinct and occurs at multiple levels. Accumulated α-syn has been shown to disrupt mitochondrial membrane integrity, interfere with vesicular trafficking, and impair mitochondrial dynamics through effects on proteins such as Mfn2 and Drp1. These alterations compromise the ability of mitochondria to maintain membrane potential and structural integrity, conditions required for efficient PINK1 stabilization and Parkin recruitment (Hou et al., 2023). In cellular and animal models with elevated α-syn, defective Parkin translocation and reduced ubiquitin signaling have been observed, indicating suppression of mitophagic activation (Bagnoli et al., 2025).

This bidirectional relationship is therefore functionally asymmetric: mitochondrial dysfunction promotes α-syn aggregation, while aggregated α-syn feeds back to inhibit mitochondrial quality control (Li et al., 2021; Shen and Dettmer, 2024). Together, these processes support the concept of a proteotoxic axis in which impaired mitochondrial clearance and α-syn pathology become increasingly interconnected, creating conditions that can reinforce cellular stress (Figure 3).

### 5.2 Mitochondrial dynamics and organelle coupling: from structural regulation to degradative failure

Efficient mitophagy requires coordinated regulation of mitochondrial dynamics and inter-organellar communication (Van Laar and Berman, 2009). The PINK1/Parkin pathway plays a central role in these processes by regulating mitochondrial fission–fusion balance and facilitating the segregation of damaged organelles (Lim et al., 2012; Liao et al., 2024).

When this pathway is impaired, mitochondrial dynamics become dysregulated. Altered activity of proteins such as Drp1 and Mfn2 leads to defective fragmentation and isolation of damaged mitochondrial segments, reducing the efficiency with which these organelles can be targeted for degradation (Van Laar and Berman, 2009). At the same time, disruption of mitochondria–lysosome contact sites interferes with local metabolic communication. Parkin has been implicated in maintaining amino acid homeostasis at these interfaces, and its dysfunction compromises the ability of lysosomes to respond to mitochondrial stress (Peng et al., 2023).

Lysosomal competence represents an additional constraint (Tofaris, 2012). Defects in lysosomal ion channels, including TMEM175, impair lysosomal pH regulation and degradative capacity (Qu et al., 2022). Under these conditions, even when mitochondria are successfully labeled, autophagic flux cannot be efficiently completed. Experimental studies have shown that impairment of lysosomal function leads to accumulation of ubiquitinated mitochondria, indicating a failure at the level of terminal degradation rather than signal initiation.

These combined defects establish a state in which mitochondrial damage is recognized but not resolved. The resulting accumulation of partially processed organelles increases cellular stress and provides a substrate for further pathological amplification.

### 5.3 Neuroinflammation as a feedback driver of mitophagy failure

When damaged mitochondria are not efficiently cleared, mitochondrial components such as mitochondrial DNA (mtDNA) and mitochondrial ROS (mtROS) can be released into the cytosol and extracellular space (Ahmed et al., 2026). These molecules act as damage-associated molecular patterns (DAMPs) that activate innate immune pathways.

mtDNA released into the cytosol engages the cGAS–STING pathway, leading to transcriptional activation of inflammatory genes through NF-κB signaling (Mun et al., 2025). In parallel, mitochondrial stress can promote activation of the NLRP3 inflammasome, resulting in maturation and release of pro-inflammatory cytokines. These pathways are not strictly independent; rather, they represent partially overlapping responses to mitochondrial damage that together sustain inflammatory signaling (Yan et al., 2023; Lv et al., 2025; Ahmed et al., 2026).

Neuroinflammation, in turn, feeds back onto mitochondrial quality control. Inflammatory mediators and oxidative/nitrosative stress can disrupt mitochondrial membrane potential and induce post-translational modifications of PINK1 and Parkin, reducing their functional activity (Oh et al., 2017). In experimental models, sustained inflammatory signaling has been associated with impaired mitophagic flux and reduced mitochondrial clearance capacity.

Through this feedback, mitochondrial dysfunction is converted into a self-reinforcing pathological loop. Impaired clearance promotes DAMP release, DAMP-driven inflammation suppresses mitophagy, and reduced mitophagy further increases the burden of damaged mitochondria.

This feedback relationship may be particularly relevant under conditions in which mitochondrial labeling and mitochondrial elimination become partially uncoupled. Under such circumstances, mitochondria that enter the mitophagy pathway but are not efficiently degraded may persist as a source of DAMPs, including mtDNA and mtROS, thereby sustaining activation of cGAS–STING and NLRP3-associated inflammatory signaling despite evidence of pathway engagement (Pereira et al., 2023; Mun et al., 2025; Ahmed et al., 2026). This possibility further reinforces the distinction between pathway activation and successful completion of mitochondrial clearance.

These interactions place mitochondrial dysfunction, *α*-syn aggregation, lysosomal impairment, and neuroinflammation within a common pathological framework. Rather than acting as separate processes, they become functionally coupled once PINK1/Parkin-dependent clearance is compromised, so that damage accumulation increasingly drives further failure of mitochondrial quality control. In this context, PINK1/Parkin pathway imbalance may function as more than an isolated defect, as impaired mitochondrial quality control can influence multiple interacting processes that contribute to the broader pathogenic network of PD (Narendra and Youle, 2024).

## 6 Therapeutic strategies targeting the PINK1/Parkin pathway

Current therapeutic efforts targeting the PINK1/Parkin pathway are driven by a central problem in PD research: mitochondrial quality control can often be stimulated experimentally, yet restoration of durable neuronal protection remains difficult to achieve (Antico et al., 2025). As summarized in Table 4, existing strategies fall into three groups—direct pathway activators, deubiquitylase (DUB) inhibitors, and interventions that improve mitochondrial or lysosomal homeostasis. Their shared goal is to enhance mitophagy-related function, but they differ in how directly they engage the pathway and in their ability to restore mitochondrial clearance.

**Table 4:** Representative therapeutic strategies targeting the PINK1/Parkin pathway and their reported neuroprotective effects in experimental PD models.

Therapeutic category	Representative agent/strategy	Experimental model and major phenotypic/behavioral outcomes	Principal molecular effects
Direct pathway enhancement	BL-918 (Wang et al., 2024)	MPTP mouse model: nigral DA neuron survival ↑; motor function ↑	PINK1 activation/accumulation ↑; p-S65-Ub ↑
	FB231 (Rosencrans et al., 2025)/MTK458 (Rosencrans et al., 2025)	HeLa cells/rat primary neurons: clearance of damaged mitochondria ↑; ATP levels ↑	Mitophagy activation threshold ↓; stress sensitivity ↑
USP30 inhibitor	MTX115325 (Fang et al., 2023)/MF-094 (Fang et al., 2023)	MPTP α-syn mouse model: nigrostriatal degeneration ↓; motor impairment ↓	USP30 activity ↓; mitochondrial ubiquitination ↑; pS129-α-syn ↓
	CMPD-39 (Rusilowicz-Jones et al., 2022)	SH-SY5Y cells: accumulation of damaged mitochondria ↓; mtROS ↓	Mitochondrial ubiquitin labeling ↑; lysosomal degradation efficiency ↑
USP25 inhibitor	AZ1 (Xu et al., 2026)	A53T mouse model: motor decline ↓; pathological protein aggregation ↓	USP25 activity ↓; mitophagic flux restoration ↑
Mitophagic flux and homeostasis optimization	NL-1 (Martinez et al., 2024)/rosiglitazone (Martinez et al., 2024)	Drosophila Pink1/parkin: climbing ability ↑; lifespan ↑	Cisd protein accumulation ↓; mitochondrial cristae structural damage ↓
	UBC9 overexpression (Liu et al., 2025)	MPTP mouse model: nigral DA neuron survival ↑; DAT levels ↑	PINK1 stability ↑; SUMO1 modification signaling ↑
AMPK-mediated indirect modulation	Dexmedetomidine (Chen et al., 2022)	MPTP mouse model: gait coordination ↑; DA fiber density ↑	AMPK phosphorylation ↑; PINK1/Parkin expression ↑
	Teaghrelin (Jhuo et al., 2024)	MPTP mouse model: nigral DA neuron number ↑; mitochondrial network complexity ↑	Mitochondrial clearance ↑; mitochondrial biogenesis ↑
	Cytarabine (Li et al., 2024)	Rat primary DA neurons: synaptophysin (SYP) expression ↑; synaptic degeneration ↓	AMPK activity ↑; clearance efficiency of damaged mitochondria ↑
Broad mitochondrial homeostasis improvement	Epicoccin A (Ye et al., 2025)	Zebrafish PD model: swimming distance ↑; DA neuron survival ↑	LC3-II/I ratio ↑; oxidative damage ↓
	Uncaria rhynchophylla alkaloid extract (URA; Hou et al., 2025)	6-OHDA rat model: tremor symptoms ↓; neuronal apoptosis ↓	cytochrome C release ↓; Caspase-3 activation ↓
Formulation and delivery system optimization	HA-But nanoparticles (Sun et al., 2026)	MPTP mouse model: motor endurance ↑; neuroinflammation ↓	Butyrate bioavailability ↑; central α-syn aggregation ↓

↑, Elevated level, upregulated expression, or functional improvement; ↓, reduced level, downregulated expression, or attenuated pathological damage. MPTP, 1-methyl-4-phenyl-1,2,3,6-tetrahydropyridine; p-S65-Ub, ubiquitin phosphorylated at serine 65; mtROS, mitochondrial reactive oxygen species; DAT, dopamine transporter; SYP, synaptophysin; LC3, microtubule-associated protein 1 light chain 3; 6-OHDA, 6-hydroxydopamine; URA, Uncaria rhynchophylla alkaloid extract.

Direct activators and DUB inhibitors provide the clearest proof that pharmacological manipulation of the pathway is feasible (Fang et al., 2023). In particular, strategies targeting USP30 are supported by coherent mechanistic evidence, as they favor sustained mitochondrial ubiquitin labeling without requiring complete reconstruction of the signaling cascade (Rusilowicz-Jones et al., 2022). By contrast, interventions that act indirectly through redox control, bioenergetic support, or lysosomal homeostasis appear more dependent on cellular context. Their effects may still be biologically meaningful, but they are better understood as modulators of the environment in which mitophagy operates than as direct restorers of PINK1/Parkin function.

A more critical issue emerges from compounds described as pathway activators. In some cases, increased PINK1/Parkin readouts do not reflect genuine recovery of mitochondrial quality control, but rather stress sensitization. This is important for agents such as FB231 and MTK458, which can lower the apparent threshold for mitophagy activation yet also impose mitochondrial stress and activate the integrated stress response (Rosencrans et al., 2025). Under these conditions, stronger p-S65-Ub signaling or enhanced Parkin recruitment cannot be taken as straightforward evidence of therapeutic rescue. The pathway may appear more active precisely because mitochondria have become more vulnerable. This distinction matters because it exposes a recurrent interpretive trap in the field: amplification of upstream signaling can be mistaken for restoration of functional mitophagy.

The broader pattern in Table 4 points in the same direction. Many interventions are evaluated primarily through upstream indicators—PINK1 accumulation, Parkin translocation, or enhanced ubiquitin signaling—whereas much less evidence is available for complete mitochondrial degradation, preserved bioenergetic competence, or durable protection of vulnerable DA neurons (Maestro et al., 2021). Incomplete coupling between signal induction and terminal clearance may represent a major mechanistic contributor to the translational gap observed between experimental and clinical studies.

The limited clinical translation of these strategies likely reflects more than the early developmental status of the field. Major obstacles include the lack of robust target-engagement biomarkers in humans, unresolved challenges in achieving brain and neuron-specific delivery, and the possibility that downstream degradative competence is already compromised by the time intervention is initiated. Under such conditions, increasing upstream signaling alone may be insufficient to generate functionally effective mitophagy, even when pathway-associated readouts appear favorable in preclinical models.

For this reason, the therapeutic value of PINK1/Parkin-targeted strategies should not be judged solely by how strongly they activate the pathway. The more important question is whether they restore functional continuity across the mitophagic sequence, from damage sensing and ubiquitin labeling to autophagosome maturation and lysosomal degradation. Without that continuity, enhanced signaling may represent little more than amplified pathway engagement under stress. Effective disease-modifying intervention will likely require not only raising upstream activity, but also re-establishing the downstream conditions that allow damaged mitochondria to be fully cleared.

## 7 Translational research on the PINK1/Parkin pathway: critical issues and clinical pathways

### 7.1 Pathway activation does not equate to functional restoration

A central obstacle in translating PINK1/Parkin-targeted strategies into clinical benefit lies in a conceptual mismatch between pathway activation and mitochondrial quality control (Narendra and Youle, 2024). Increased PINK1 stabilization, enhanced Parkin recruitment, and elevated ubiquitin signaling are often taken as evidence of pathway restoration. Yet these upstream events mark only the initiation of mitophagy and do not, by themselves, demonstrate that damaged mitochondria are successfully cleared (Lazarou et al., 2015).

Functionally effective mitophagy depends on coordinated progression through multiple downstream steps, including cargo recognition, autophagosome maturation, long-range transport in neuronal processes, and lysosomal degradation (Tofaris, 2012). Interruption at any of these stages can produce a state in which mitochondria are labeled but not eliminated (Pereira et al., 2023; Zagare et al., 2025). The interpretation of such outcomes is further complicated by the fact that increased pathway-associated signals may arise either from improved signaling efficiency or from stress-driven threshold crossing (Rosencrans et al., 2025). These two scenarios can generate superficially similar readouts while implying very different biological consequences.

### 7.2 Toward an operational definition of activation–clearance uncoupling

This distinction becomes particularly relevant in the context of the PINK1/Parkin pathway. Quantitative analysis of the mitophagy decision circuit suggests that pathway activation requires mitochondrial PINK1 to exceed a critical threshold generated through positive-feedback amplification (Waters et al., 2023). Crossing this threshold is therefore necessary for pathway engagement. However, mitochondrial turnover requires progression through multiple downstream steps that are not directly governed by the activation threshold itself (Klionsky et al., 2021). As a result, activation of the pathway does not necessarily guarantee efficient mitochondrial clearance.

Several observations suggest that pathway engagement and degradative output do not always remain tightly coupled. In neuronal systems, initiation, trafficking, and degradation frequently occur in distinct cellular compartments and on different timescales (Lu et al., 2026). Likewise, studies of aging and neurodegeneration have reported accumulation of p-S65-Ub-positive structures that are not uniformly associated with complete mitochondrial elimination (Hou et al., 2018; Richardson et al., 2025). These findings support the possibility that mitochondrial labeling and mitochondrial removal may become partially dissociated under specific pathological conditions, and are consistent with the activation–clearance uncoupling framework proposed in this Review.

**Figure 4:**
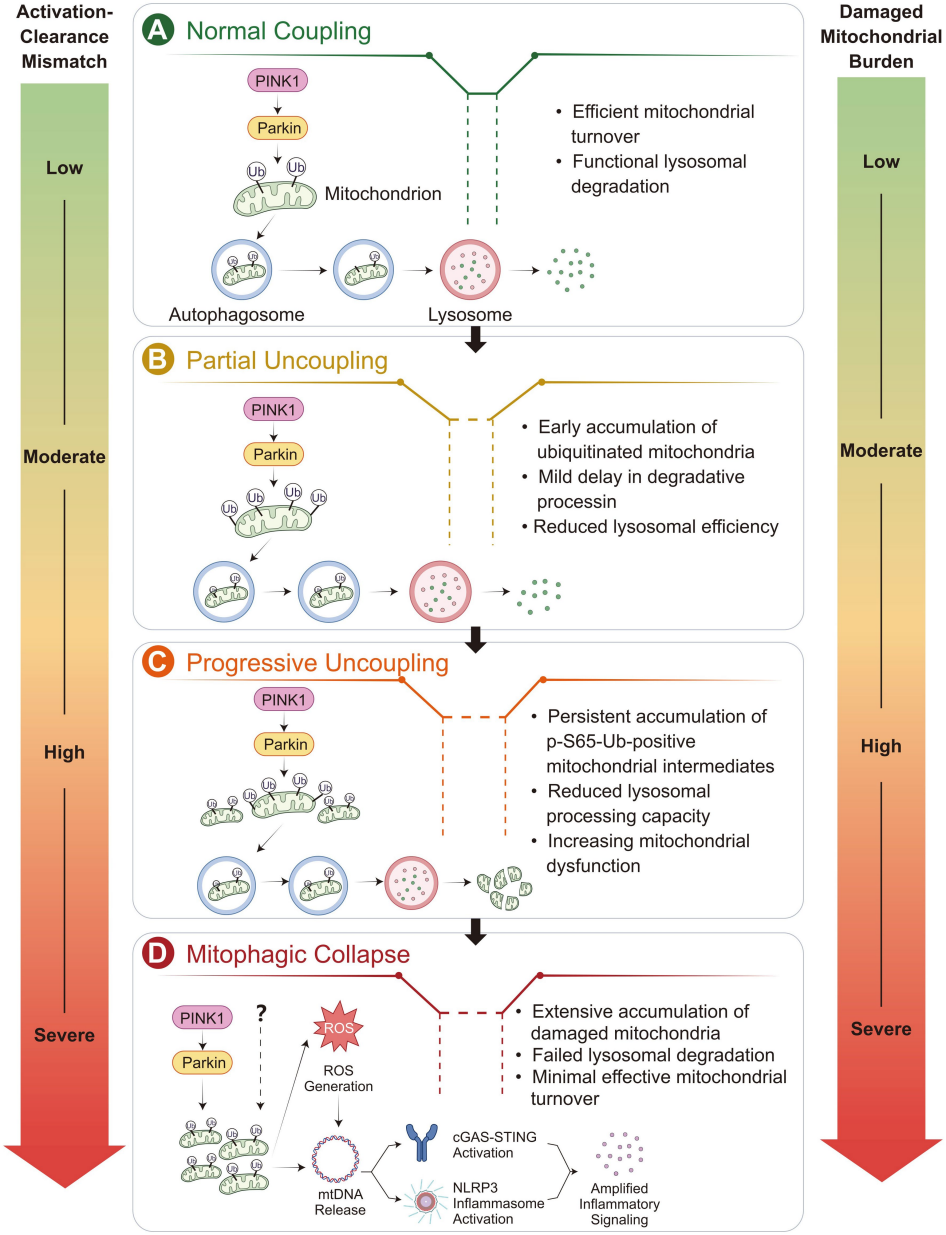
Activation–clearance continuum framework. **(A)** Normal coupling: pathway engagement and mitochondrial clearance remain coordinated, supporting efficient mitochondrial turnover. **(B)** Partial uncoupling: clearance efficiency begins to decline despite detectable pathway engagement, resulting in early accumulation of ubiquitinated mitochondria and reduced lysosomal efficiency. **(C)** Progressive uncoupling: persistent pathway engagement is accompanied by accumulation of p-S65-Ub-positive mitochondrial intermediates, impaired degradative capacity, and increasing mitochondrial dysfunction. **(D)** Mitophagic collapse: severe failure of mitochondrial turnover leads to accumulation of damaged mitochondria, oxidative stress, release of mitochondrial danger-associated signals, and activation of inflammatory pathways, including cGAS–STING and NLRP3 signaling. The widening mismatch zone represents increasing dissociation between pathway engagement and effective mitochondrial clearance, whereas damaged mitochondrial burden progressively increases across the continuum.

We use the term activation–clearance uncoupling to describe this mismatch and to organize the operational criteria discussed below, while recognizing that this framework is intended to generate testable predictions rather than to assert a mechanism that has been fully characterized. Operational features that may support this interpretation are summarized in Table 5, whereas broader conceptual distinctions from insufficient activation are outlined in Table 6. Importantly, activation–clearance uncoupling should not be viewed as a strictly binary state. Rather, the relationship between pathway activation and mitochondrial clearance is more appropriately considered as a continuum of progressively impaired coupling efficiency, as illustrated in Figure 4.

**Table 5:** Diagnostic criteria for distinguishing activation–clearance uncoupling from insufficient activation.

Domain	Marker	Insufficient activation	Activation–clearance uncoupling	Representative evidence
Upstream activation status	Activation threshold	Low	High	Waters et al. (2023)
	PINK1 stabilization	Reduced	Present	Lazarou et al. (2015) and Waters et al. (2023)
	Parkin recruitment	Reduced	Present	Lazarou et al. (2015)
	p-S65-Ub signal	Low	High/partial	Hou et al. (2018) and Watzlawik et al. (2024b)
Downstream clearance status	Mitochondrial ubiquitination	Low	High/partial	Lazarou et al. (2015)
	Autophagosome recruitment	Reduced	Partial	Klionsky et al. (2021) and Vargas et al. (2023)
	Lysosomal competence	Present	Reduced/partial	Qu et al. (2022)
	Mitophagic flux	Low	Reduced	Vargas et al. (2023) and Shojaei et al. (2025)
	Mitochondrial turnover	Low	Reduced/partial	McWilliams et al. (2016) and Vargas et al. (2023)
Pathological consequences	Mitochondrial intermediates	Rare	Frequent	Richardson et al. (2025)
	Damaged mitochondrial burden	Low	High	Hou et al. (2018)
	Neuroinflammation	Low	Partial/high	Moehlman et al. (2023) and Park et al. (2025)

Some markers (e.g., p-S65-Ub, mito-QC, mt-Keima, mitophagic flux) are laboratory-based and not suitable for clinical diagnosis. This table provides a conceptual framework for research. Representative references are listed.

**Table 6:** Operational interpretation of activation–clearance uncoupling.

Question	Insufficient activation	Activation–clearance uncoupling
Pathway engaged?		
Clearance completed?	No	No
Primary defect	Initiation failure	Downstream processing failure
Functional continuity	Not established	Disrupted

Conceptual assessment; no literature references included.

At present, no quantitative threshold defines when activation becomes uncoupled from clearance. One reason is that pathway activation and mitochondrial turnover are typically assessed using different experimental readouts and often on different biological timescales. Consequently, a standardized metric linking pathway engagement to degradative output has not yet been established. Future studies integrating activation markers, lysosomal function, and direct measurements of mitochondrial turnover may help identify biologically meaningful transition points along this continuum. Until such approaches become standardized, activation–clearance uncoupling is best considered a working framework for interpreting discordant mitophagy findings rather than a formally validated disease mechanism.

The interpretation of pathway-associated biomarkers requires distinction between evidence of pathway engagement and evidence of mitochondrial turnover. As summarized in Table 2, commonly used markers such as p-S65-Ub, PINK1, and Parkin provide information regarding pathway-associated signaling but do not directly quantify mitophagic flux. Consequently, identical biomarker changes may be compatible with multiple biological states, including increased pathway activation, impaired downstream clearance, or mixed responses involving both processes. Dynamic measurements remain the preferred strategy for distinguishing pathway initiation from pathway completion, although their application in human clinical studies remains limited (McWilliams et al., 2016; Morinaga et al., 2024; Fiesel et al., 2025). A practical next step will be the development of longitudinal and human neuron–based systems capable of linking pathway-associated signaling to actual mitochondrial elimination.

### 7.3 Future directions and validation priorities

A central challenge for future studies is to move from pathway-associated observations toward operational criteria that define when activation and clearance become dissociated. One potential approach is the development of quantitative activation-to-clearance metrics that integrate pathway-associated signaling with independent measurements of mitochondrial turnover. Such metrics may help determine whether Activation–Clearance Uncoupling represents a graded continuum or a discrete transition occurring beyond a biologically meaningful threshold.

This challenge is closely linked to the interpretation of clinical biomarkers. As discussed above and summarized in Table 2, current evidence is strongest for pathway activation and substantially weaker for direct assessment of mitochondrial clearance. Future studies may therefore benefit from combining p-S65-Ub, PINK1, or Parkin measurements with longitudinal sampling, lysosomal function assessments, and turnover-sensitive approaches capable of capturing temporal changes in mitochondrial fate rather than static molecular states.

A related opportunity lies in the development of human neuron–based experimental systems that could extend current mechanistic observations to more disease-relevant contexts. Human induced pluripotent stem cell–derived dopaminergic neurons, together with dynamic approaches such as mito-QC, mt-Keima, or related turnover-based assays, may provide practical platforms for examining the relationship between pathway activation and mitochondrial elimination under disease-relevant conditions, and for testing whether the activation–clearance framework applies across different genetic and cellular contexts (McWilliams et al., 2018a; Morinaga et al., 2024). Such approaches may be particularly valuable because they enable assessment of mitochondrial turnover dynamics and therefore help distinguish pathway activation from successful mitochondrial clearance.

The most important unresolved question concerns sporadic PD. Current evidence supports the disease relevance of mitochondrial quality-control abnormalities but does not establish that the activation–clearance relationship observed in genetic PD generalizes to the broader PD population (Hou et al., 2018; Hong et al., 2024; He, X. et al., 2025). Future studies combining pathway-associated biomarkers, dynamic flux measurements, and patient-derived neuronal systems may help evaluate whether similar mechanisms operate across both genetic and sporadic forms of the disease, and could provide a basis for refining the activation–clearance framework in clinically relevant contexts.

### 7.4 Experimental models and readouts introduce systematic bias

Translational inconsistency is amplified by limitations built into the experimental systems most commonly used to study mitophagy (Kaya and Kırboğa, 2026). Acute toxin-based models, overexpression systems, and genetic loss-of-function models all remain informative, but they do not distort the pathway in the same way (Klionsky et al., 2021; Tran et al., 2026). Acute toxin exposure favors abrupt mitochondrial depolarization and strong upstream activation. Overexpression systems increase apparent pathway responsiveness by elevating protein abundance beyond physiological levels. Genetic models are valuable for defining causality, yet their phenotypes may also reflect compensatory or developmental adaptation.

Despite these differences, the models converge on a common problem: they tend to make upstream signaling easier to observe than downstream clearance failure. This bias is reinforced by the readouts that are most often used. Measurements such as PINK1 accumulation, Parkin translocation, or p-S65-Ub levels are highly sensitive indicators of pathway engagement, but they provide only limited information about whether mitophagy has been completed (Watzlawik et al., 2024a).

In neurons, this gap becomes even harder to ignore (Cason et al., 2022). Mitophagy unfolds across extended cellular compartments, and the timing of autophagosome formation, retrograde transport, and lysosomal fusion can vary substantially between soma and distal processes. Under these conditions, short-term or compartment-restricted measurements may overestimate pathway activity while missing stalled intermediates or delayed degradative failure.

A more informative evaluation framework should therefore separate pathway engagement from pathway completion within the same experimental system. In this context, p-S65-Ub accumulation or Parkin recruitment can be used to indicate upstream signal initiation, whereas persistence of ubiquitinated mitochondrial intermediates helps identify stalled progression (Sarraf et al., 2013; Tolosa et al., 2026). Measures of lysosomal competence are needed to assess terminal degradative capacity, and mitochondrial clearance kinetics or morphological evidence of organelle turnover provide the closest approximation to completed mitophagy (Qu et al., 2022). Used in combination rather than isolation, these readouts can distinguish effective clearance from a state in which signaling is activated but degradation remains incomplete. Without this kind of integration, experimental outcomes are prone to conflate signal amplification with functional restoration.

### 7.5 Clinical translation requires alignment of timing, stratification, and endpoints

The same interpretive problems become more consequential in the clinical setting, where patient populations are heterogeneous with respect to genetic background, disease stage, and the relative contribution of mitochondrial, lysosomal, and inflammatory pathology (Rigon et al., 2026). A pathway-targeted intervention may therefore enter biologically distinct contexts across patients who share the same clinical diagnosis.

Timing is one major source of divergence (Foltynie et al., 2026). Early in disease, when mitochondrial and lysosomal competence are only partially compromised, increasing pathway responsiveness may still improve organelle turnover. Later in the disease course, the same intervention may have little effect if downstream degradative capacity has already deteriorated or if substantial neuronal loss has occurred. This is why earlier intervention matters in two separate senses: it means treating at an earlier biological stage of disease, but it also means targeting earlier mechanistic failure points within the mitophagic sequence, before downstream clearance becomes irreversibly compromised.

Patient selection presents a related challenge. Stratification cannot rely only on broad clinical phenotype or single biomarker positivity (Tolosa et al., 2026). It needs to reflect which part of the pathway is most impaired in a given patient population—threshold crossing, signal amplification, or downstream clearance. Only then can pathway-targeted therapies be matched to a biological context in which they remain capable of producing a functional effect.

Outcome measures must also be aligned with pathway biology. Global motor scales and late clinical endpoints remain important, but they are poorly suited to detecting early changes in mitochondrial quality control. More informative trial designs will likely require biologically anchored endpoints, including dynamic mitophagy-related biomarkers, molecular measures of pathway engagement interpreted in context, and, where feasible, imaging-based assessments of mitochondrial or lysosomal function (Hey et al., 2025). None of these measures is likely to be sufficient on its own, but together they offer a more realistic basis for evaluating whether pathway-directed interventions produce sustained biological benefit.

The limited clinical success of current strategies does not necessarily argue against the relevance of the PINK1/Parkin pathway itself (Wang et al., 2023). It more likely reflects a persistent mismatch between experimental readouts, patient selection, trial timing, and the actual biology of mitochondrial quality control in PD. Effective translation will depend on aligning these elements so that pathway activation is measured in a way that remains biologically meaningful and functionally sustained.

## 8 Discussion

The synthesis of current evidence suggests that dysfunction of the PINK1/Parkin pathway in PD is more accurately understood as a failure of coordination within a multistep mitochondrial quality control system, rather than a simple reduction in pathway activity (Narendra and Youle, 2024). This perspective helps reconcile a persistent inconsistency in the field: pathway-associated signals are frequently robustly activated in experimental models and, in some cases, detectable in patient-derived systems, yet the relationship between such activation and sustained restoration of mitochondrial function or clinical benefit has not been clearly established.

Throughout this Review, Activation–Clearance Uncoupling has been used as an interpretive framework to integrate observations that are not readily explained by a strictly linear view of mitophagy. As summarized in Table 2, support for this framework derives primarily from mechanistic studies demonstrating that pathway activation and mitochondrial elimination can be experimentally dissociated, whereas current clinical evidence relies largely on static biomarker measurements that do not directly capture mitophagic flux. The available data therefore support the plausibility of Activation–Clearance Uncoupling as an integrative framework, while also identifying areas where further empirical characterization would strengthen its interpretive scope. Accordingly, this framework is best regarded as a conceptual model for integrating current observations and generating testable hypotheses.

At a mechanistic level, the PINK1/Parkin pathway operates as a threshold-dependent, feed-forward system capable of amplifying mitochondrial damage signals (Waters et al., 2023). However, successful mitophagy depends on the continuity of this signal through downstream processes, including organelle segregation, autophagosome maturation, intracellular transport, and lysosomal degradation (Horowitz et al., 2022). When these downstream steps are compromised, a pathological state emerges in which mitochondria are effectively labeled but incompletely cleared (Lazarou et al., 2015). This uncoupling between signal initiation and terminal degradation provides a unifying explanation for why increased pathway activity does not necessarily correspond to improved mitochondrial homeostasis.

This interpretation stands in contrast to more conventional views that primarily attribute pathway dysfunction to insufficient activation or loss-of-function mutations. While these mechanisms are clearly relevant—particularly in familial PD—they do not fully account for observations in sporadic disease, where pathway activation may still be detectable despite progressive neurodegeneration. In such contexts, additional layers of regulation become important. For example, post-translational modifications such as S-nitrosylation have been shown to suppress PINK1 activity under conditions of oxidative and nitrosative stress, suggesting that pathway output can be constrained even in the presence of upstream activation signals (Oh et al., 2017). These observations highlight a broader conceptual divergence in the field: whether mitophagy failure is best understood as a deficit in activation or as a breakdown in coordinated pathway execution.

Placing this problem within a broader cellular context further clarifies its implications (Pereira et al., 2023). Mitochondrial dysfunction in PD is embedded within a network that includes *α*-syn aggregation, lysosomal impairment, and neuroinflammatory signaling (Yan et al., 2023). These processes interact bidirectionally and reinforce one another, creating conditions in which both damage accumulation and clearance failure are progressively amplified. Within this network, impairment of the PINK1/Parkin pathway functions less as an isolated defect and more as a destabilizing factor that both contributes to and is exacerbated by system-wide dysfunction (Sironi et al., 2020). This perspective helps explain why interventions targeting single molecular nodes often fail to produce durable effects.

The same considerations extend to therapeutic development. As outlined in preceding sections, a large proportion of candidate interventions enhance upstream readouts such as PINK1 stabilization, Parkin recruitment, or ubiquitin signaling, yet show inconsistent effects on mitochondrial turnover. In some cases, apparent pathway activation may arise from increased mitochondrial stress rather than improved quality control, raising the possibility that enhanced signaling reflects sensitization rather than restoration (Rosencrans et al., 2025). Among current therapeutic concepts, inhibition of deubiquitylases such as USP30 may represent one of the most mechanistically coherent strategies for enhancing mitochondrial ubiquitin signaling and facilitating downstream mitophagic processes (Fang et al., 2023). Such approaches may better support mitophagic flux by sustaining ubiquitin labeling independently of acute mitochondrial stress. Whether this translates into durable clearance improvement remains an open question (Rusilowicz-Jones et al., 2022).

These therapeutic considerations should also be interpreted within a broader biological context. Pathway-associated biomarkers measured in peripheral biofluids or postmortem tissue cannot directly determine whether mitochondrial clearance is impaired within vulnerable nigral dopaminergic neurons, because such readouts capture selected aspects of mitochondrial quality control rather than the complete degradative process. Disease stage is likely to be equally important. Interventions that improve mitochondrial turnover may be more effective before extensive neuronal loss and lysosomal dysfunction become established, whereas later-stage disease may be less responsive even when pathway-associated signaling remains detectable (Foltynie et al., 2026). In addition, Parkinson’s disease is increasingly recognized as a biologically heterogeneous disorder in which mitochondrial dysfunction may contribute differently across genetic and sporadic subtypes (Rigon et al., 2026). Basal mitochondrial turnover can also involve complementary quality-control mechanisms beyond canonical PINK1/Parkin signaling, indicating that disruption of this pathway should be interpreted within a broader mitochondrial quality-control network (McWilliams et al., 2016). These considerations do not argue against the Activation–Clearance Uncoupling framework. Rather, they help define the biological context in which uncoupling may emerge and influence therapeutic responses.

A related challenge concerns the interpretation of biomarkers. Peripheral measurements of pathway-associated signals, including p-S65-Ub or circulating PINK1 levels, provide accessible indicators of pathway engagement but do not reliably distinguish between effective mitochondrial clearance and accumulation of partially processed intermediates (Baninameh et al., 2025). This limitation becomes particularly relevant in clinical translation, where reliance on upstream biomarkers may overestimate therapeutic impact. More informative assessment strategies will likely require integration across multiple levels, combining molecular readouts with functional measures of mitochondrial turnover and, where feasible, imaging-based approaches that capture organelle dynamics *in vivo* (Zhu et al., 2024).

Several key gaps remain. The conditions under which pathway activation becomes uncoupled from mitochondrial clearance are not yet well defined and may differ across neuronal subtypes, disease stages, and genetic backgrounds. Quantitative relationships between upstream signaling dynamics and downstream degradation efficiency remain poorly characterized (Norouzi Esfahani et al., 2025), limiting the interpretability of commonly used experimental and clinical readouts. In addition, the extent to which partial restoration of pathway function is sufficient to produce meaningful clinical benefit is still unclear. Addressing these questions will require experimental systems capable of resolving the temporal and spatial progression of mitophagy, as well as longitudinal studies linking molecular changes to functional outcomes (Kaya and Kırboğa, 2026).

These considerations point toward a shift in research and therapeutic strategy. Rather than focusing primarily on increasing pathway activity, future approaches should aim to restore functional continuity across the mitophagic process. This includes not only enhancing damage sensing and ubiquitin signaling, but also supporting downstream processes such as mitochondrial dynamics, intracellular transport, and lysosomal degradation. At the clinical level, improved alignment between patient stratification, intervention timing, and biologically relevant outcome measures will be essential for accurately assessing therapeutic efficacy.

The PINK1/Parkin pathway is best viewed not as a linear signaling cascade but as a component of a dynamic, context-dependent quality control network (Pereira et al., 2023). Reframing the problem in this way provides a coherent explanation for past inconsistencies and offers a more realistic foundation for the development of disease-modifying strategies in PD.
